# p53 Requires the Stress Sensor USF1 to Direct Appropriate Cell Fate Decision

**DOI:** 10.1371/journal.pgen.1004309

**Published:** 2014-05-15

**Authors:** Amine Bouafia, Sébastien Corre, David Gilot, Nicolas Mouchet, Sharon Prince, Marie-Dominique Galibert

**Affiliations:** 1 CNRS-UMR6290 Genetic and Development Institute of Rennes, GEO Team, Rennes, France; 2 Université de Rennes 1, UEB, SFR Biosit, Rennes, France; 3 University of Cape Town, Department of Human Biology, Faculty of Health Sciences, Cape Town, South Africa; 4 CHU Rennes, Service de génétique moléculaire et génomique, Rennes, France; University of Washington, United States of America

## Abstract

Genomic instability is a major hallmark of cancer. To maintain genomic integrity, cells are equipped with dedicated sensors to monitor DNA repair or to force damaged cells into death programs. The tumor suppressor p53 is central in this process. Here, we report that the ubiquitous transcription factor Upstream Stimulatory factor 1 (USF1) coordinates p53 function in making proper cell fate decisions. USF1 stabilizes the p53 protein and promotes a transient cell cycle arrest, in the presence of DNA damage. Thus, cell proliferation is maintained inappropriately in *Usf1* KO mice and in USF1-deficient melanoma cells challenged by genotoxic stress. We further demonstrate that the loss of USF1 compromises p53 stability by enhancing p53-MDM2 complex formation and MDM2-mediated degradation of p53. In USF1-deficient cells, the level of p53 can be restored by the re-expression of full-length USF1 protein similarly to what is observed using Nutlin-3, a specific inhibitor that prevents p53-MDM2 interaction. Consistent with a new function for USF1, a USF1 truncated protein lacking its DNA-binding and transactivation domains can also restore the induction and activity of p53. These findings establish that p53 function requires the ubiquitous stress sensor USF1 for appropriate cell fate decisions in response to DNA-damage. They underscore the new role of USF1 and give new clues of how p53 loss of function can occur in any cell type. Finally, these findings are of clinical relevance because they provide new therapeutic prospects in stabilizing and reactivating the p53 pathway.

## Author Summary

Cancer is a complex disease that is characterized by the sequential accumulation of genetic mutations. Exposure to environmental agents, such as solar ultraviolet, induces such alterations and thus contributes to the development of genomic instability. The tumor suppressor p53 has a central role in orchestrating cellular responses to genotoxic stress. In response to DNA-damage, p53 is stabilized and activated to direct cell fate decisions. Cells in which p53 stabilization is compromised become more vulnerable to mutagenic agents and hence the mutation rate increases, which promotes tumor development. Stabilization of p53 is thus a critical step towards cancer prevention. Using a genetic approach, we demonstrate that the ubiquitous transcription factor Upstream Stimulatory factor 1 (USF1) is required for immediate p53 stabilization and appropriate cell fate decisions following genotoxic stress. Furthermore, we show that this involves a novel function of USF1 that underscores its critical role as a stress sensor. The loss of USF1 expression should thus be considered as a potential initiator of tumorigenesis in the context of environmental insults.

## Introduction

Genomic instability is a central hallmark of cancer, where DNA damaging agents play an important role [1]–[2]. The transformation of normal cells into cancer cells requires the succession of several genetics alterations within the genome that alter key physiological regulatory processes.

In DNA-damaged eukaryotic cells, genome integrity is maintained by an immediate and inducible protective program. This program requires dedicated sensors that drive and regulate the cellular response, by monitoring DNA-repair and if required by forcing damaged-cells into cell death pathways [3]. When these sensors are compromised, sensitivity to mutagenic agents is increased and the mutation rate speeds up, allowing tumor development. The extent of DNA lesions and the capacity of dedicated sensors to direct a proper response are thus determining parameters of cell fate.

To date, the tumor suppressor p53 is the most important sensor [4], being a central and early regulator of the DNA-damage response. Upon recognition of DNA damage, p53 induces a transient growth arrest by holding the cell cycle at the G1/S regulation point. p53 acts through activating the expression of the cell-cycle arrest gene *CDKN1A* (*p21*) [5], [6], [7], allowing DNA repair and thereby preventing the development of cancer. This p53-dependent transient cell cycle arrest is thus a decisive step in cell fate that requires the stabilization of p53 protein. Indeed, in the absence of cellular stress, p53 is maintained at low steady-state levels by the dynamic p53-MDM2 feedback loop [8]. In response to DNA-damage signaling, the p53 protein undergoes extensive post-translational modifications including phosphorylation by DNAPK, ATM and ATR, all members of the PI3K family [9]. These modifications nucleate subsequent changes in the repertoire of proteins interacting with p53 and in particular abolish the p53-MDM2 interaction [10]. This results in the immediate increase in p53 levels and transcriptional activity, thereby directing cell fate decisions [11], [12], [13].

The Upstream Stimulatory Factor 1 (USF1) is an ubiquitous transcription factor of the basic-helix-loop-helix leucine-zipper (bHLH-LZ) family that operates as a stress sensor. USF1 is a direct target of the p38 stress-activated kinase and genetic studies demonstrate that USF1 is a transcriptional rheostat for the stress response [14], [15], [16]. In response to UV-radiation, a physiological source of direct DNA-damage, known as the major risk factor for skin cancers [17], [18], [19], [20], USF1 regulates the expression of pigmentation genes [15], and genes of the nucleotide excision repair pathway (NER) [21]. This protective function of USF1 is important since the repair of DNA damage is central to the maintenance of genome stability.

The USF1 and p53 pathways both have pivotal roles in the response to stress, where they participate in the immediate molecular and cellular responses. They regulate common biological processes to mitigate deleterious effects. Both pathways have been studied in detail, but little is known about any crosstalk between them, although *in vitro* studies suggested that USF1 may regulate the basal transcription of the *p53* gene [22], [23]. We thus examined whether USF1 could contribute to the canonical p53 stress response by directing proper cell fate decisions. We used a combination of *in vivo* and *in vitro* genetic approaches to test for the presence of a coordinated USF1/p53 program. We demonstrate that in the presence of DNA damage, USF1 is necessary for immediate p53 protein stabilization and that the p53-mediated cell cycle arrest requires USF1. We report evidence that USF1 is a central regulator of p53 to direct cell fate decisions, identifying thereby a new functional and unexpected role for USF1. Collectively, these findings have important and broad consequences for our understanding of mechanisms that maintain stress-induced DNA damage and cancer promotion.

## Results

### USF1-deficient mouse skin is unable to up-regulate p53 in presence of DNA damage

**Figure 1:**
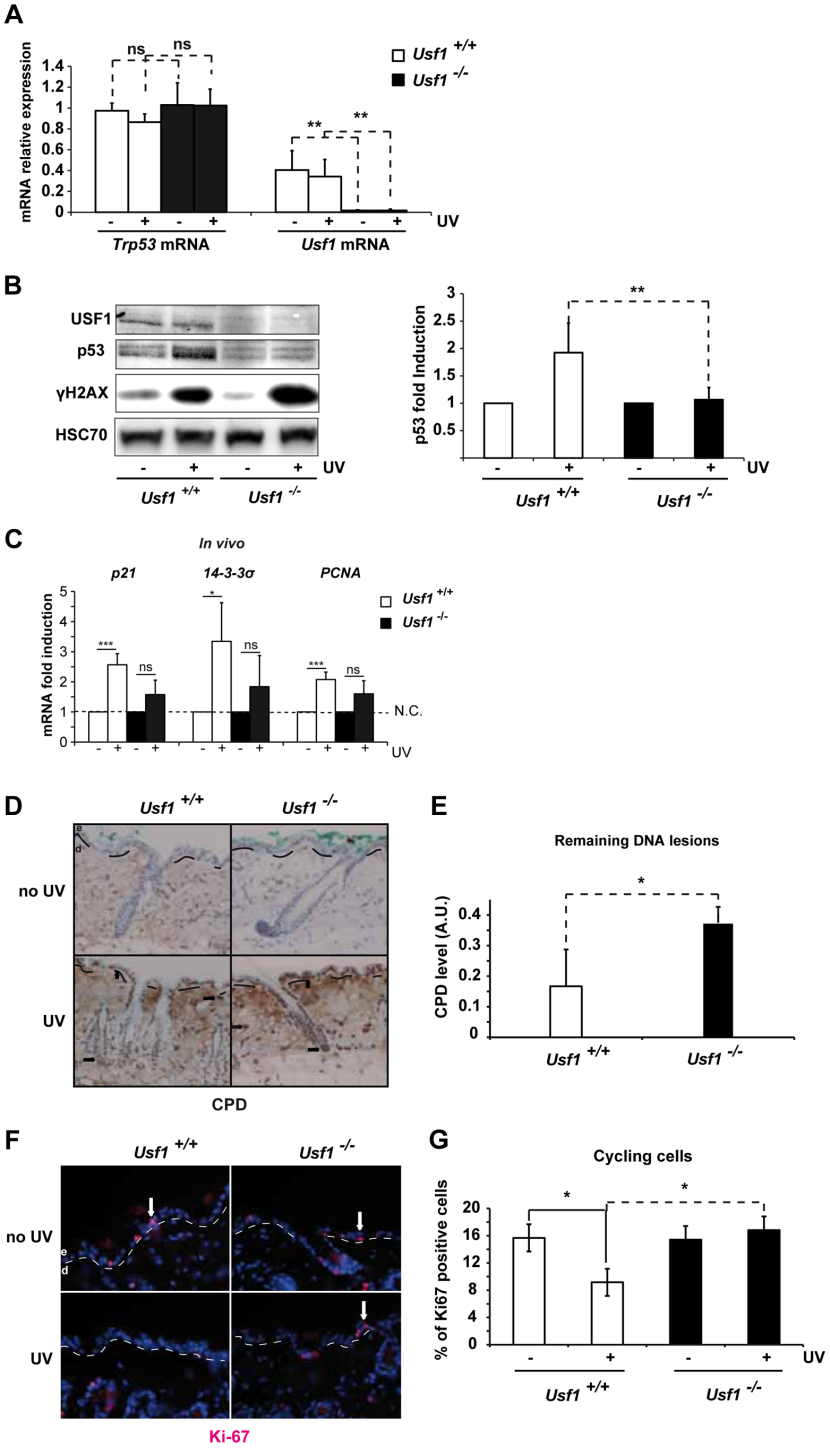
*Usf1* KO mice present defective induction of p53 protein. The back of *Usf1* KO mice (*Usf1^-/-^*) and WT mice (*Usf1^+/+^*) were irradiated or not irradiated with an UVB dose corresponding to the mice MED (5 kJ/m^2^) and the skin was analyzed 5 h later. (A) RT-qPCR analysis of *Trp53* and *Usf1* mRNA relative level (expressed as a ratio to the value for the *Hprt* transcript) in skin extracts from protected (-) and UV-exposed (+) areas. Error bars: SD, n>9. (B) Western blot showing USF1, p53, γH2AX and HSC70 (loading control) immunoreactivity 5 h after skin irradiated or not irradiated with UVB. The graph reports the mean ratio between the p53 signal (normalized to that for HSC70) in skin-exposed areas versus non-irradiated areas (controls). Error bars: SD, n = 8 for each condition. (C) *Usf1^+/+^* (Usf1 WT) and *Usf1^-/-^* (*Usf1* KO) skins were or were not irradiated with UVB (5 kJ/m^2^) and analyzed for the induction of transcripts *in vivo.* RT-qPCR analysis of *CDKN1a* (p21), *SFN* (14-3-3σ) and *PCNA* transcripts in UVB-irradiated skin and non-exposed controls; values reported were normalized to those for the *Hprt* transcript. Transcripts were assayed *in vivo* 5 hours after irradiation. Error bars: SD, n = 4 *in vivo* (D) Immunohistochemical labeling of cyclobutane pyrimidine dimers (CPD) showing their localization and abundance in skin areas (x100) exposed or not exposed to UVB. Dashed lines indicate the boundary between the dermis (d) and the epidermis (e), and arrows indicate positive nuclei. (E) The level of CPDs in total DNA extracts from skin was quantified by ELISA. The graph shows the mean difference in the CPD absorbance values between for exposed and protected skin areas. Error bars: SD, n = 4. (F) Immunofluorescence staining with the Ki-67 antibody of inter-follicular cycling cells in skin areas (x100) exposed or not exposed to UVB. (G) The graph shows the mean percentage of cycling cells (calculated as Ki-67-positive cells/total Dapi-stained cells) in protected and UV-exposed skin areas. Error bars: SD, n = 3. Student's *t* test was used to test the significance of differences (*, *p* <0.05, **, p<0.01, ***, p<0.001).

To identify a coordinated USF1/p53 program, we first examined p53 expression (by assaying mRNA and protein levels) and the p53 acute stress response in *Usf1* ^-/-^ mice. Mice were challenged with UVB irradiation, a physiological inducer of direct DNA-damage, known to activate the p53 pathway [24]. We quantified *Trp53* mRNA in skin cells from *Usf1* KO mice and WT littermates (n = 9 for each genotype) and found no significant differences between the two genotypes both before and 5 hours after UVB radiation (Figure 1A). Similarly, the basal level of the p53 protein was low, with no statistical difference (Wilcoxon Mann-Whitney test with W = 0,98) between the two genotypes (n = 16 and n = 11 for respectively *Usf1* KO mice and WT littermates). However, while a significant and reproducible 2-fold increase of the p53 protein was observed in WT littermates 5 hours post-UVB irradiation, p53 protein-levels remained low and unchanged in *Usf1* ^-/-^ mice (Figure 1B). Phosphorylation of the H2AX histone (γH2AX), a substrate of the DNAPK/ATM/ATR axis [25], [26], increased following irradiation in both genotypes confirming comparable signal transmission of UVB-induced DNA damage (Figure 1B). Levels of p53 remained low in *Usf1* ^-/-^ mice compared to their WT littermates 12 h post-irradiation (Figure S1A). This ruled out the possibility that the p53 response in *Usf1* ^-/-^ mice was simply delayed. Following UVB-irradiation, the *p21*, *14-3-3 sigma* and *PCNA* genes were less strongly induced in *Usf1* ^-/-^ than control mouse skin both *in vivo* (*Usf1* ^-/-^ mouse skin; Figure 1C) and *ex vivo* (*Usf1* ^-/-^ cultured skin biopsies; Figure S1B). Thus, the absence of induction of p53 in the *Usf1* ^-/-^ mice was accompanied by weaker up-regulation of some p53 target genes required for the DNA-damage response, 5 hours post-irradiation. In addition, and in accordance with the use of the mice minimal erythema dose (MED), *Bax* and *Puma* pro-apoptotic genes were not up-regulated 5 hours post-irradiation in both genotypes (data not shown).

*Trp53*-deficient mice have reduced DNA repair ability and impaired cell cycle arrest in response to DNA-damaging agents [27], [28]. We therefore used immunohistochemistry (IHC) to examine the effect of USF1 deficiency on these processes. Levels of cyclobutane pyrimidine dimers (CPDs) in the epidermis and dermis and in the bulge region, 5 hours post-irradiation, were higher in *Usf1* ^-/-^ mice than WT littermates (Figure 1D). This was confirmed by ELISA, which showed that there was twice as much CPD in *Usf1* ^-/-^ mouse skin (5 h post-UV; n = 4, p<0.05) (Figure 1E). We next examined the proliferation index of epidermal cells by IHC using Ki-67, the cellular marker of cycling cells [29]. In non UV-exposed skin, the proliferation index in the inter-follicular areas was comparable in the two genotypes. In response to UVB irradiation, however, the proliferation index remained constant in *Usf1* ^-/-^ mice whereas it decreased by approximately 50% in WT littermates (Figure 1, F and G). The defect of DNA repair (Figure S1C and S1D) and the absence of cell cycle control (Figure S1E) in reponse to UVB was also observed in cultured skin biopsies of *Usf1* ^-/-^ mice, up to 24 h after irradiation. Thus, in addition to defective induction of p53 protein upon UVB exposure, *Usf1* deficient cells fail to down-regulate their cell cycle despite the presence of DNA damage.

### USF1 is required for p53-dependent G1/S arrest upon genotoxic stress

**Figure 2:**
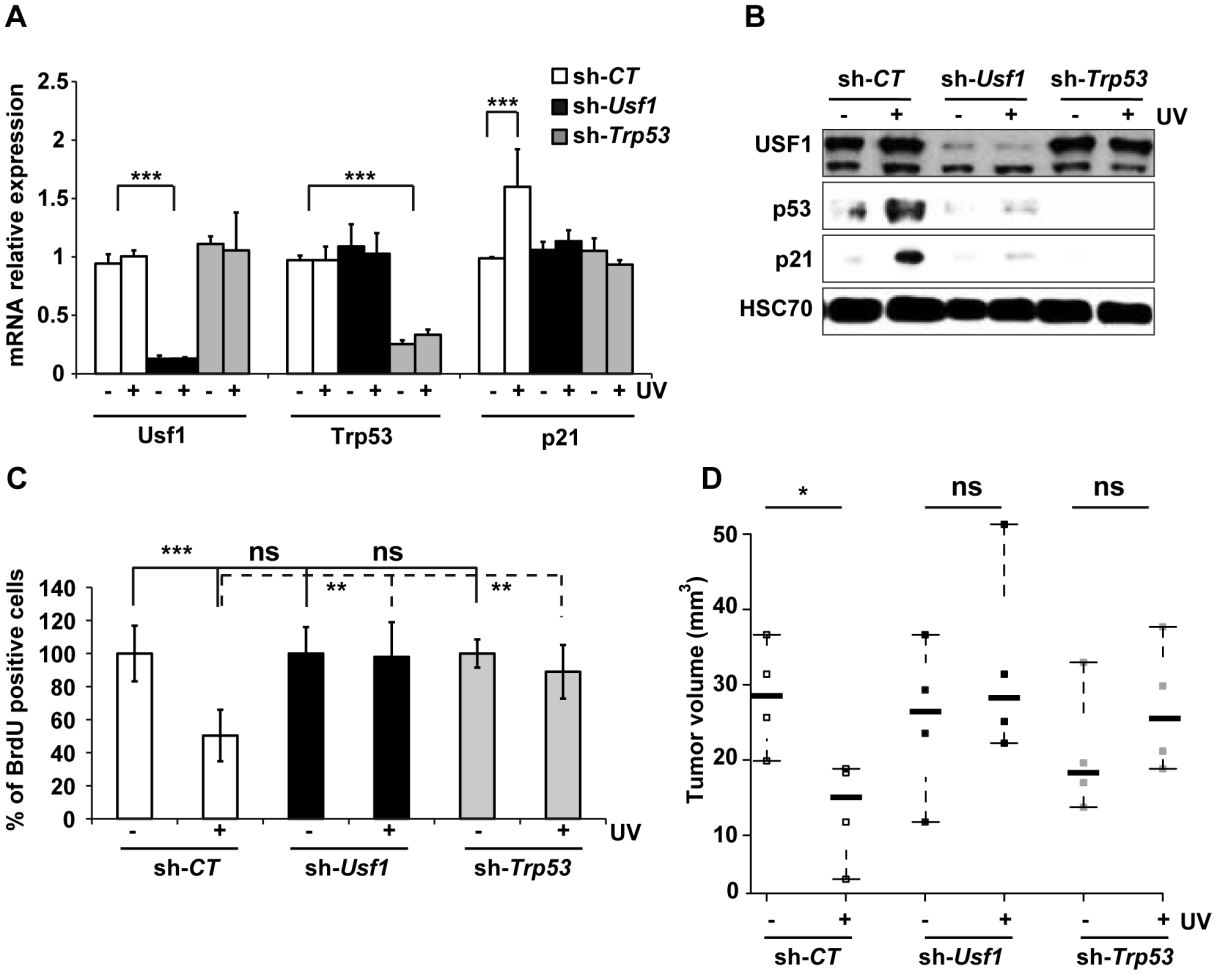
USF1 mediates p53-dependent cell cycle arrest. B16 melanoma cells knocked down for *Usf1* (sh-*Usf1*) or p53 (sh-*Trp53*) and control cells (sh-*CT*) were synchronized in G1/early S phase. The cells were then irradiated or not irradiated with UVB (0.3 kJ/m^2^) and the cell cycle released. (A) *Trp53*, *Usf-1* and *p21* mRNAs in cells irradiated (+) or not irradiated (-) with UVB were quantified by RT-qPCR 3 hours after the release of the cell cycle; results are reported relative to the values for the *Hprt* transcript. Error bars: SD, n = 3. (B) Western blot analysis of USF1, p53, p21 and HSC70 (loading control) in protein extracts from cells treated as in A. (C) BrdU incorporation assay in cells irradiated or not irradiated with UVB. The values plotted are mean percentages of BrdU incorporating cells after UVB irradiation compared to those for non-irradiated cells. Error bars: SD, n = 3. (D) Stripchart plot showing the volume of tumors formed 12 days after subcutaneous injections of 2.10^4^ B16 melanoma cells (sh-*Usf1*, sh-*Trp53* or sh-*CT*). UVB (0.3 kJ/m^2^) irradiated or control cells, for which cell viability had been controled and was identical, were injected into the two sides of the back of NOD/SCID mice. Error bars: SD, n = 4 for mice injected with sh-*CT* and sh-*Trp53*, and n = 5 for mice injected with sh-*Usf1* cells. Student's t test was used for statistical analysis (*, *p* <0.05, **, p<0.01, ***, p<0.001).

To decipher the specific contribution of USF1 and p53 proteins to the regulation of cell cycle progression upon genotoxic stress, we generated stable knock-down (KD) cell lines using the B16 mice melanoma cells that express active p53 and USF1 pathways. The effectiveness of the shRNAs used to knock down *Usf1* and *Trp53* was verified (Figure 2, A and B). Levels of *Trp53* mRNA were comparable in *Usf1* KD and control cells (sh-*CT*) and remained unchanged in response to UVB, whereas the levels of the p53 protein increased only in UVB-irradiated control cells (Figure 2, A and B). The mRNA and protein levels of p21, the p53-dependent effector of the G1/S arrest, remained low in both *Usf1* KD and *Trp53* KD cells in response to UVB, whereas they increased in control cells. Furthermore, consistent with findings for *Usf1* ^-/-^ mice, time course experiments showed that there was no delayed UV-induced p53 and p21 up-regulation in *Usf1* and *Trp53* KD cells (Figure S3A). These findings showed that the KD cell culture models reproduced features of *Usf1* ^-/-^ mice. To examine S phase progression upon genotoxic stress, cells were synchronized and we followed the synthesis of DNA by measuring the incorporation of a thymidine analogue (BrdU). The results show that the proliferation rates of synchronized *Usf1* and *Trp53* KD cells were similar to that of control cells (Figure 2C). However, in the UVB-irradiated condition, while the number of BrdU-incorporating cells remained unchanged and comparable for the *Usf1* and *Trp53* KD cells, irradiated control cells exhibited a significant reduction, of 50%, in the number of BrdU-incorporating cells. Similar results were obtained using primary fibroblasts isolated from *Usf1^-/-^* mice and *Usf1^+/+^* littermates (Figure S2). These data are consistent with the *in vivo* results (Figure 1, F and G), highlighting a general mechanism. In addition, USF1 levels did not differ between *Trp53* KD cells and controls, indicating that USF1 expression is not dependent on p53 (Figure 2, A and B). This also suggests that the deficiency in cell cycle arrest of *Usf1* KD cells in response to genotoxic stress may be the result of the absence of increased levels and/or activity of p53 and p21 [5], [6], [7], [30], [31].

The loss of p53 is a critical event that promotes tumor growth. We therefore investigated whether loss of USF1 favors tumor growth *in vivo* under stress conditions. To this end, we injected NOD/SCID mice subcutaneously with mock- or UVB-irradiated, viable *Usf1* and *Trp53* KD cells, and examined tumor growth 12 days later. The tumors produced by UVB-irradiated control cells were half the size of those produced by mock-irradiated control cells (Figure 2D). *Usf1* and *Trp53* KD cells both generated massive tumors and their sizes were not modified by UV-pretreatment (Figure 2D). This demonstrates that USF1, like p53, is required for the transient cell cycle arrest in order to delay cell proliferation in response to induced DNA damage.

### USF1 is critical for p53 protein stabilization

**Figure 3:**
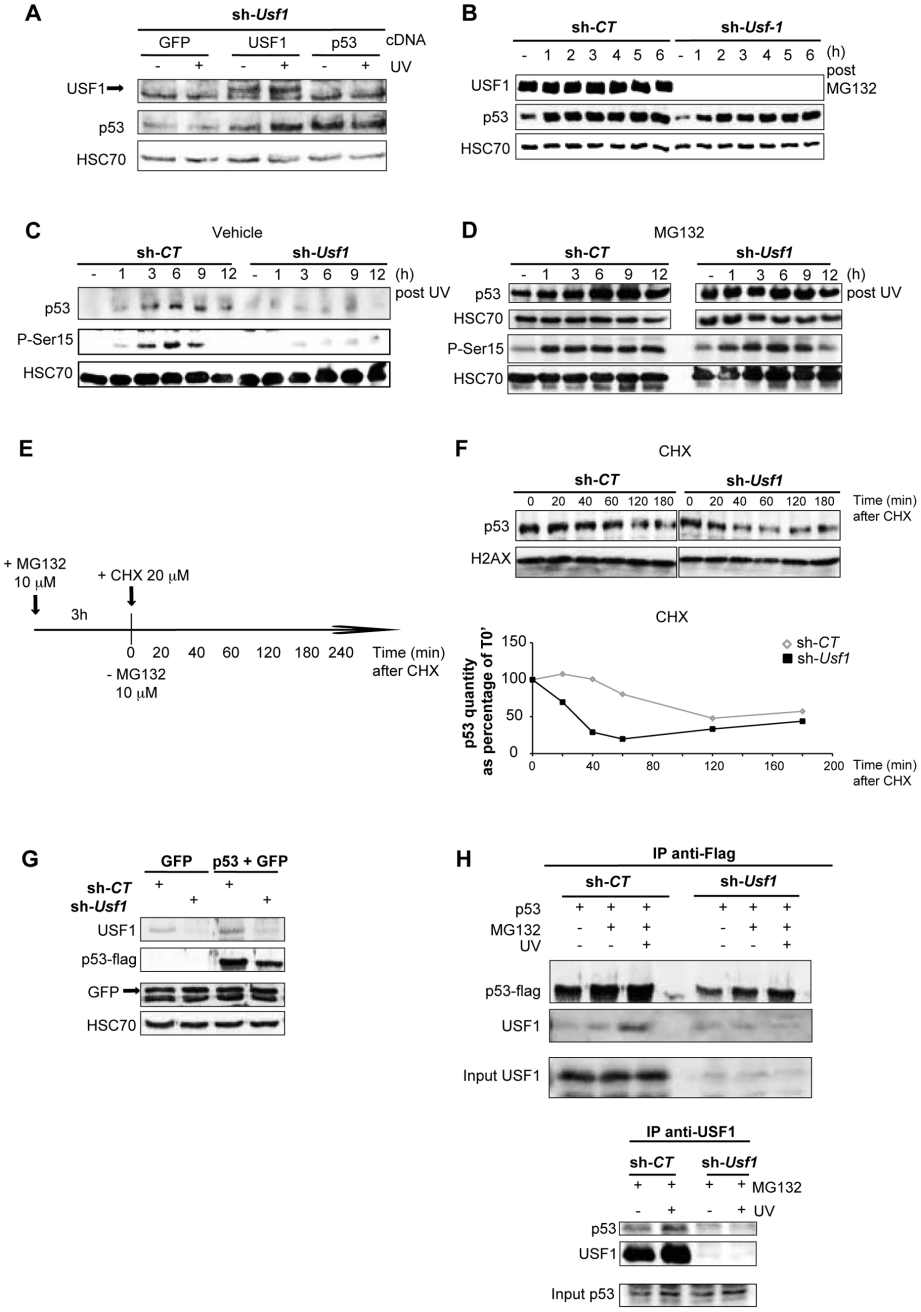
USF1 is required to stabilize p53 protein following genotoxic stress. B16 melanoma cells knocked down for *Usf1* (sh-*Usf1*) and their controls (sh-*CT*) were analyzed for post-translational regulation of p53. (A) Western blot analysis of the effect of USF1 re-expression on p53 protein levels in sh-*Usf1* cells irradiated or not irradiated with UVB and tested 6 h after irradiation. Cells were transfected with the cDNA indicated (as described in the materials and methods) and analyzed for USF1, p53 and HSC70 (loading control). (B) Western blot showing USF1, p53 and HSC70 immunoreactivity in sh-*CT* and sh-*Usf1* cells at the indicated time following treatment with MG132 (10 µM). (C–D) Time course of p53 accumulation and Ser15-phosphorylation in sh-*CT* and sh-*Usf1* cells treated with vehicle (DMSO) in C or MG132 (10 µM) plus UVB (0.3 kJ/m^2^) irradiation in D. (E–F) p53 degradation in sh-*CT* and sh-*Usf1* cells pretreated for 3 h with MG132 (10 µM) and then with cycloheximide (CHX 20 µM) (E). Cells were analyzed at the time points indicated after addition of CHX. The graphs show the results of densitometric analysis of p53 immunoreactive bands (normalized to the loading controls H2AX or HSC70). (G) Western blot showing flag-tagged p53 and GFP proteins in sh-*CT* or sh-*Usf1* cells after co-transfection of the corresponding cDNA. (H, upper panel) Immunoprecipitation analysis to assay flag-tagged p53 after transfection of sh-*CT* or sh-*Usf1* with the corresponding cDNA. Cells were treated with MG132, were or were not irradiated with UVB and analyzed 3 hours later. (H, lower panel) sh-*CT* and sh-*Usf1* cells were treated with 10 µM MG132 for 3 hours then irradiated or not irradiated with UVB. Western blot analysis of proteins immunoprecipitated from cell lysates with USF1 antibody and blotted with p53 (1C12) and USF1 antibodies.

We next investigated how USF1 controls p53 protein levels. USF1 was re-expressed in *Usf1* KD cells and we showed that this restored the induction of p53 protein (Figure 3A) and p53 transcriptional activity in response to stress (Figure S3B). The effects of re-expressing USF1 were independent of *Trp53* transcript levels (data not shown) and similar results were obtained with USF1 mutants lacking the DNA binding domain as well as the transcriptional activation domain (Figure S3C). These observations suggest that USF1 positively regulates p53 protein levels and activity independently of its transcription factor function. Therefore, USF1 may act through translational and/or post-translational mechanisms to modulate p53 availability. Treatment of *Usf1* KD and control cells with MG132 (an inhibitor of proteasome activity) resulted in immediate and similar increases of p53 protein levels in the two types of cell lines (Figure 3B). This indicates that USF1 prevents the degradation of p53 rather than inducing p53 synthesis. Furthermore, the abundance of USF1 protein in control cells remained unchanged when proteasome activity was inhibited (Figure 3B), validating the use of the MG132 inhibitor as a powerfull *in vitro* tool to further investigate the mechanism of p53 stabilization in the *Usf1* KD background.

Phosphorylation of p53 is important for its stabilization and is dependent on the activation of the DNA damage signal transducers, DNAPK, ATM and ATR. Since the phosphorylation of serine 15 (Ser15) in the p53 protein is required to mediate interactions with other proteins to block contact with its inhibitor, MDM2 [32], [33], we specifically examined this modification. *Usf1* KD and control cells were pre-treated with vehicle or MG132 to stabilize the p53 protein and exposed to UVB. In the absence of MG132 pre-treatment, UVB-induced phosphorylation of Ser15 and stabilization of p53 occurred only in control and not in *Usf1* KD cells (Figure 3C). Inhibition of the proteasome degradation pathway in the presence UVB resulted in comparable levels of phosphorylated Ser15 and stabilization of p53 in *Usf1* KD cells and control cells (Figure 3D). These results, together with data showing that phosporylation of Chk1, a downstream target of the ATM/ATR pathway implicated in p53 activation [34], is maintained in *Usf1* ^-/-^ mice (Figure S3D) and in the *Usf1* KD cells in response to UV (Figure S3E). This suggests that while upstream mechanisms of transduction of the DNA-damage signal, targeting p53-stabilization, are functional in *Usf1* KD cells, the absence of USF1 prevents full stabilization of p53.

We next examined whether USF1 modulates the half-life of p53. Cells were pre-treated with MG132 (for 3 hours) to stabilize p53, and time course experiments were performed with the protein translation inhibitor, cycloheximide (CHX) (Figure 3E). The half-life of the p53 protein in *Usf1* KD cells was 30 min, and in control cells was 110 min (sh-*CT*) (Figure 3F). To confirm these results *Usf1* KD and control cells were co-transfected with a vector encoding a flag-tag p53 construct and a GFP control construct. GFP was expressed at the same level in the two cell lines, but p53 levels in *Usf1* KD cells were half that in control cells (Figure 3G). These *in vitro* results together with work from the Levine group [35], [36] suggest that the steady state level of p53 depends on the experimental systems used (ie cell tranfection, chemical compound), which are known to challenge cells. We next examined the half-life of p53 by irradiating cells before CHX addition and our results show that the half-life of p53 was over 180 min in control cells but only 60 min in *Usf1* KD cells (Figure S4A). Together this supports a role for USF1 in modulating the half-life of p53 under conditions of stress.

To examine whether impairment of p53 stabilization could be associated with the binding of USF1 with p53, overexpressed flag-tag p53 was immuno-precipitated from both *Usf1* KD and control cells transfected as above (Figure 3G) and treated with or without MG132 and UVB. We observed an interaction of p53 with USF1 only in control cells and this interaction is notably increased after UV irradiation when the p53 protein is stabilized (Figure 3H, upper panel). In order to confirm this interaction between p53 and USF1, we performed immunoprecipitations assays with USF1 antibody in *Usf1* KD and control cells, pre-treated with MG132 and following exposure to UVB. Again, only in the presence of USF1 was an interaction observed between USF1 and p53 which was particularly evident after UV irradiation (Figure 3H, lower panel). These results highlight the potential function of the USF1 transcription factor in stabilizing the p53 protein through a direct interaction.

### USF1 associates with p53 and inhibits MDM2-mediated p53 degradation

**Figure 4:**
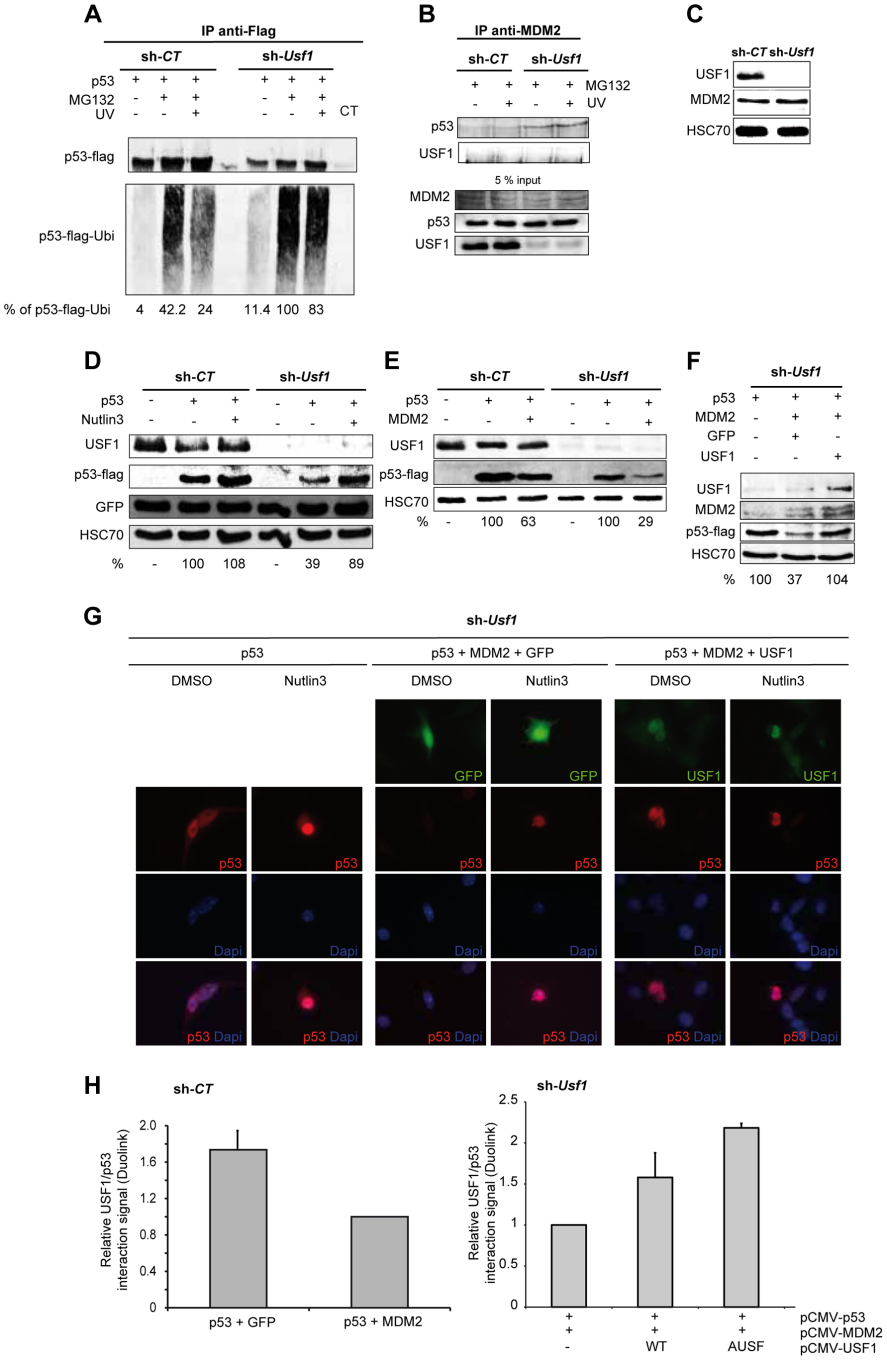
USF1 counteracts MDM2-mediated p53 degradation upon cellular stress. p53 protein-protein interactions and MDM2 mediated p53 degradation were studied in B16 melanoma cells knocked down for *Usf1* (sh-*Usf1*) and their controls (sh-*CT*). (A–B) sh-*CT* and sh-*Usf1* cells were treated with 10 µM MG132 for 3 hours then irradiated or not irradiated with UVB. Western blot analysis of proteins immunoprecipitated from cell lysates (A) Immunoprecipitation analysis to assay ubiquitinated flag-tagged p53 after transfection of sh-*CT* or sh-*Usf1* with the corresponding cDNA. Cells were treated with MG132, were or were not irradiated with UVB and analyzed 3 hours later. The values reported indicate the level of p53 ubiquitination (normalized to the total amount of flag-tagged protein recovered). p53 expressing sh-*Usf1* cells treated with MG132 has been arbitrary chosen as the reference (100%) since it is the condition where normalized-level of p53-ubiquitinated protein is the highest. (B) sh-*CT* and sh-*Usf1* cells were treated with 10 µM MG132 for 3 hours then irradiated or not irradiated with UVB. Western blot analysis of proteins immunoprecipitated from cell lysates with a mix of two MDM2 antibodies (SMP14 and 3G9) and blotted with p53 antibody (1C12). (C) Western blot analysis showing basal levels of USF1, MDM2 and HSC70 (loading control) in sh-*CT* and sh-*Usf1* cell lysates. (D) Western blot showing the effect of Nutlin-3 (10 µM, 6 h) treatment on the levels of flag-tagged p53 and GFP proteins in sh-*CT* and sh-*Usf1* cells; antibodies to USF1, p53, GFP and HSC70 (loading control) were used. (E) Western blot analysis of p53, MDM2 and HSC70 (loading control) in sh-*CT* and sh-*Usf1* cells over-expressing either p53 or p53 plus MDM2. (F) Same experiment as in D but in sh-*Usf1* KD cells over-expressing either GFP or USF1. (G) Immunofluorescence analysis of p53 expression and localization in sh-*Usf1* KD cells treated as in D and stimulated with vehicle (DMSO) or Nutlin-3 (10 µM) for 6 hours. Experiments have been done in triplicate and 15 to 20 microscopic fields analyzed per condition. (H) Quantification of the level of p53 and USF1 interaction in B16 melanoma cells using Thermo Scientific Cellomics HCS Solution (fluorescent microscopy) using Duolink PLA technology. Quantification of p53-USF1 interaction level using specific primary antibodies and Duolink PLA technology in B16 melanoma sh-*CT* cells over-expressing either p53 or p53 plus MDM2 (left panel). The graph represents the cumulative level of fluorescence observed in B16 cells under specific spotted form. p53 plus GFP is used as control condition. Quantification of p53-USF1 interaction level in B16 melanoma sh-*Usf1* cells over-expressing p53 plus MDM2 and or not different forms of USF1 (wild type or negative dominant (AUSF)) (right panel). p53 plus MDM2 is used as control condition.

Since stabilization of p53 in response to genotoxic-stress is dependent on the regulation of its proteasomal degradation, we measured the rate of p53-ubiquitination in the absence of USF1. The basal level of ubiquitinated flag-tag p53 was approximately three times higher in *Usf1* KD than control cells (Figure 4A). Following MG132 treatment there was a substantial accumulation of ubiquitinated flag-tag p53 in *Usf1* KD cells. Irradiation following MG132 treatment had almost no effect on the levels of ubiquitinated flag-tag p53 in *Usf1* KD cells but this level was almost half in control cells (Figure 4A). These investigations demonstrate that USF1 interferes with the process of p53 ubiquitination and thereby maintains p53 stability following exposure to genotoxic agents.

MDM2 is the E3-ubiquitin ligase that interacts with p53 to promote p53 degradation by the proteasome and is therefore a central regulator of p53 stability [8]. We thus examined whether USF1 protects p53 from interacting with MDM2 and consequently preventing its degradation, by using immunoprecipitation assays performed with antibodies to MDM2 (Figure 4B). The anti-MDM2 antibody precipitated p53 with MDM2 from *Usf1* KD cells but not from the control cells and UVB irradiation had no significant effect (Figure 4B). These results suggest that in the absence of USF1, the interaction between p53 and MDM2 is favored. These immunoprecipitation experiments showed that USF1 and MDM2 did not interact with one another. Also, MDM2 was similarly abundant in both control and knock down cell lines under all conditions tested, indicating that silencing *Usf1* did not interfere with MDM2 levels (Figure 4, B and C). In addition, we demonstrate that the level of the MDM2 protein remains unchanged under the cell culture conditions previously tested (Figure S4 B–D). Together these results suggest that USF1 and MDM2 could bind p53 in a competitive manner.

To confirm that the interaction of MDM2 was responsible for the increased degradation of p53 in *Usf1* KD cells, we examined the levels of flag-tag p53 in the presence of Nutlin-3, a specific inhibitor of the MDM2-p53 interaction (Figure 4D). Consistent with our previous results, the expression of flag-tag p53 in *Usf1* KD cells was half that in control cells. A short treatment (6 h) with Nutlin-3 almost completely restored the level of flag-tag p53 in *Usf1* KD cells. We next compared the ability of MDM2 to degrade p53 in the presence and absence of USF1. Control and *Usf1* KD cells were transiently co-transfected with vectors expressing p53 and MDM2. Whereas co-expression with MDM2 led to a decrease of approximately 70% of p53 protein in *Usf1* KD cells, there was only a 37% decrease in control cells (Figure 4E). When we restored USF1 expression, the degradation of p53 mediated by MDM2 was completly counteracted (Figure 4F). These results provide further evidence that USF1 contributes to protecting p53 from MDM2-mediated degradation.

MDM2 has been reported to promote nuclear export of p53 and thereby targetting it for degradation [35]. We therefore determined whether USF1 can interfere with MDM2 mediation of p53 cellular localization under conditions described in Figure 4F and using Nutlin-3. In the presence of Nutlin-3, the levels of p53 were exclusively nuclear and higher compared to control (vehicle). When p53 and MDM2 were co-expressed, p53 was completely degraded confirming the activity of MDM2 on p53. As expected, the presence of Nutlin-3 almost completely counteracted the MDM2-mediated degradation of p53 and led to accumulation of p53 in the nucleus. Finally, similar to the results obtained for Nutlin-3, co-expression of USF1 with p53 and MDM2 abolished p53 degradation and maintained the nuclear localization of the p53 protein (Figure 4G). Under comparable conditions of overexpression, we quantified the interaction between p53 and USF1 using the Duolink PLA techonology, and show that the number of p53/USF1 interactions decreased when MDM2 was co-expressed (Figure 4H). In addition, re-expression of USF1 (WT or AUSF forms), with p53 and MDM2 led to a significant increase of the number of p53/USF1 interactions (Figure 4H, right panel). Together, these data demonstrate that USF1 interferes with MDM2-mediated nuclear export of p53 and its subsequent degradation by directly interacting with p53.

## Discussion

**Figure 5:**
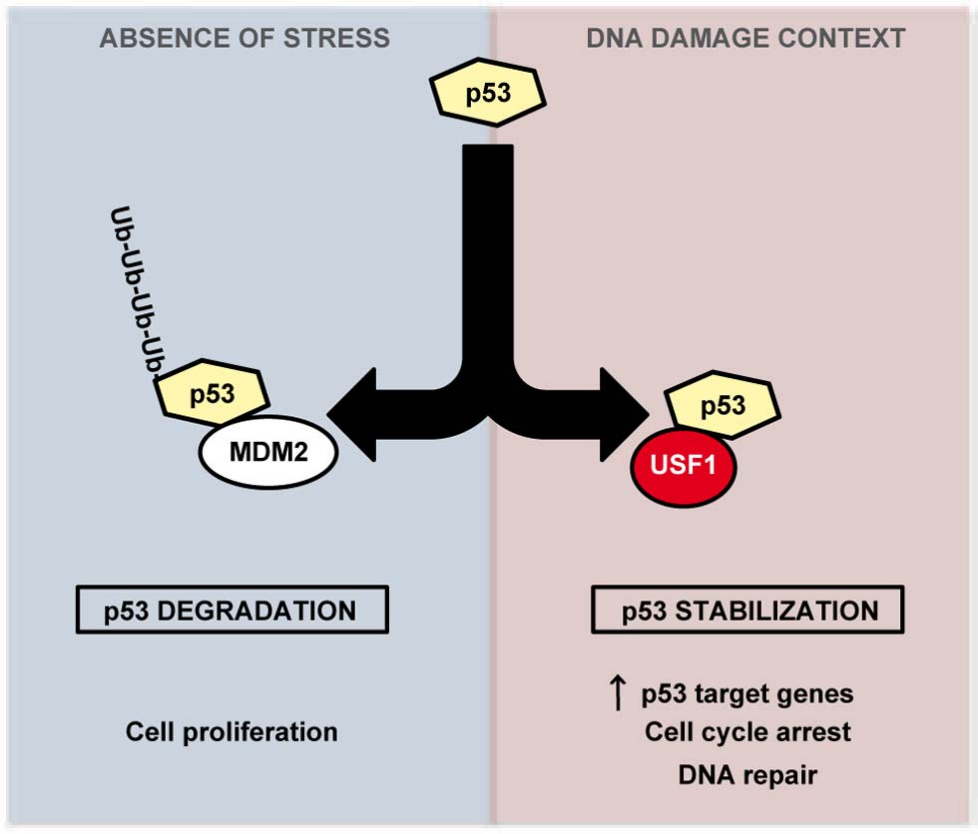
Model of regulation of p53 stabilization by USF1 in response to stress. USF1 prevents MDM2-mediated p53 degradation under stress conditions, thereby ensuring the stability and tumor suppressor activity of the p53 protein. Left Panel, in the absence of stress, p53 is targeted to proteasomal degradation after binding to MDM2, maintaining cell proliferation. Right Panel, under DNA-damage context, USF1 counteracts MDM2 function by interacting with p53 thereby increasing its transcriptional activity to control transient cell cycle arrest and DNA repair processes. In the absence of USF1, p53 stabilization is abolished abrogating cell cycle control in response to DNA damage and thereby favoring genomic instability.

Activation of the p53 pathway in response to DNA damage is a critical mechanism that selectively directs cells to transient cell cycle arrest to favor DNA repair or to promote cell death when DNA damage is irreparable. Disruption of this protective pathway leads to an increase in cells' mutation load promoting genomic instability and frequently cancer development [36]. Our study demonstrates a new role for USF1 as a key upstream regulator of the p53 pathway. We provide compelling evidence that USF1 binds to p53 under UV stress conditions, preventing MDM2-mediated p53 degradation. Under stress conditions, USF1 thereby ensures the stability and tumor suppressor activity of the p53 protein. We thus propose that USF1 directs appropriate p53-dependent cell fate decisions in response to genotoxic stress (Figure 5).

The transcription factors USF1 and p53 are both associated with the stress response and with cell proliferation [37], [38], [39] and while there is evidence suggesting a link between them, this had not been demonstrated *in vivo*. For example, USF1 and p53 regulate the transcription of common target genes including *APC*, *BRCA2* and *hTERT* [18], [40], [41], [42], [43], [44], and *in vitro* studies implicated USF1 in the basal regulation of the *Trp53* promoter [22], [23]. Here, we report that, in mice skin and in B16 melanoma cells, USF1 drives DNA damage-induced cell cycle arrest by regulating p53 protein stability and function. Thus *in vivo* loss of USF1 parallels p53 deficency [27], [45], with altered proliferation control in the presence of DNA-damage. And like *Trp53* ^-/-^ [46], [47] or *p21* ^-/-^ cells [5], *Usf1* ^-/-^ primary fibroblasts bypass the transient cell cycle arrest triggered by DNA damaging radiation. We further found that under basal conditions the skin of WT and *Usf1* ^-/-^ mice contained similar levels of p53 mRNA and protein, but observed differences in p53 protein levels between the two genotypes after UVB challenge. Despite previous suggestions, we could not demonstrate a transcriptional link between USF1 and p53 [22], [23]. This could possibly be attributed to cell specificities or stress-dependent contexts in which levels and activities of transcriptional factors, required to drive *Trp53* gene expression may vary [48]. Indeed, in studies involving human skin and keratinocytes UV-induced p53 was shown to be regulated only at the post-translational level but not at the mRNA level, while irritants that promote erythema induced p53 mRNA expression [49], [50]. These observations indicate that USF1 may act through more than one route to increase p53 levels according to cell type. In skin and in response to UV-induced DNA damage USF1 controls the p53 protein stability. This is expected to be of significant impact, since one attractive strategy for cancer therapy is based on p53 reactivation in cancers encoding normal but inactivated p53 protein, as observed frequently in melanomas [51], [52], [53].

Our evidence that USF1 stabilizes the p53 protein suggests that it may have functions independent of its well-defined role as a transcriptional regulator. Indeed, USF1 regulates gene expression through binding E-box regulatory elements in the promoters of target genes or by acting as a docking platform to recruit chromatin-modifying enzymes such as CBP/p300, PCAF, Set7/9, HDAC9 [54], [55], [56], [57], [58]. We now demonstrate that USF1 physically associates with the p53 complex. The ability of USF1 to promote p53 function appears to be independent from its ability to bind DNA. We cannot however exclude the possibility that the interaction between these two transcription factors may be important to bring p53 in the vicinity of p53 DNA-responsive element. Together this provides new prospects for how USF1 and p53 may regulate the expression of common target genes. In addition, it shows that USF1 can function through a new and unexpected mechanism to control cellular processes, broadening the role of USF1 and of members of the b-HLH-LZ transcription factors family.

Here we demonstrate that, in response to stress, USF1 associates with p53 to ensure p53 function. USF1 thereby prevents MDM2-mediated ubiquitination and subsequent degradation of p53. This mechanism relies on a stress-dependent association of USF1 with the p53 protein. Other stress-inducible transcription factors have been reported to contribute to the regulation of p53. For example, the transcription factor YY1 [59] enhances MDM2-mediated ubiquitination of p53 while ATF3 [60] prevents p53 ubiquitination and TAFII31 [61] prevents MDM2 association with p53. Although these transcription factors share a common role with USF1 in mediating p53 stability, the function of USF1 is not redundant since loss of USF, on its own, impedes p53 stabilization. The importance of USF1 in regulating p53 function may first be attributed to their hierarchical relation. *Trp53* KD cells express normal levels of USF1 but they are not able to direct cell cycle arrest as observed for *Usf1* KD cells. Similarly, overexpression of USF1 in p53-null Saos2-cells is not able to mimic the effect of p53 on cell proliferation, while USF1 promotes p53 function in p53 expressing cells [62]. USF1 is thus proposed to operate as an upstream regulator of p53 stability and function. Second, the abundance of USF1 may also support its critical role in directing p53 function. Indeed, while USF1 is constitutively expressed, ATF3 and YY1 mediated p53-interaction requires their induction in response to genotoxic stress [59], [60]. This suggests that USF1 is an immediate regulator of p53 stabilization in response to genotoxic stress. This however does not exclude the possibility that these transcription factors could act sequentially. Why and how the association of one factor with p53 is favored over another remains however to be elucidated. One possibility could be that the nature and intensity of the DNA damage regulate this to influence p53-directed cell fate [13], [63].

To date, the implication of USF1 in cancer development has been investigated through only one angle, its function as a transcription factor. SNPs affecting USF1 binding to the *Pten* promoter have been found to be associated with Cowden syndrome [64]; the loss of USF1 transcriptional activity has been described in several breast cancer cell lines [65] and the activation of the *hTERT* gene in oral tumors is associated with the decreased expression of USF1 and USF2 [66]. Together, this supports the transcriptional role of USF1 in cancer development, although no association has been reported between mutations in the *USF1* coding sequence and UV-induced cancer or other cancers [67], [68], [69]. Cancers are associated with exposure to environmental and biological carcinogens, including virus infection, tobacco smoke and sunlight, both of which promote DNA hypermethylation [70]. Interestingly, oncogenic transformation by *Helicobacter pylori* infection is associated with the methylation of the *USF1* promoter and subsequent inhibition of USF1 protein production [71]. In addition, *Helicobacter pylori* infection has been also shown to impair p53 protein stability [72]. It remains to be elucidated whether this mechanism of stress-induced epigenetic transformation contributes to silencing of USF1 and thus impairing p53 stability and whether it is a new mechanism of how p53 loss of function may occur in cancer cells.

In this work we demonstrate that USF1 is a critical stress sensor required to direct appropriate p53-dependent cell fate decisions. USF1 operates through a new and unexpected function revealing additional functions for bHLH-LZ factors. Finally, our findings suggest that the loss of USF1 expression should be consider as a potential initiator of tumorigenesis in the context of environmental insults.

## Materials and Methods

### Mouse skin irradiation

*Usf1* ^-/-^ (KO) and *Usf1^+/+^* (WT) mice were kindly provided by Sophie Vaulont (Cochin Institute) [73]. Animals 8–12 weeks old were used for UV irradiation experiments. Mice were maintained under specific pathogen-free (SPF) conditions in our accredited animal facilities (A 35 – 238 – 40). For *in vivo* irradiation, the backs of the mice were shaved, and one area was protected (non-exposed control) and another irradiated (exposed area). For *ex vivo* analysis, skin biopsies (0.8 cm diameter) were recovered from the back of WT and *Usf1* ^-/-^ mice and maintained in culture as previously described (Baron Y. et al., 2012). Skins were irradiated with a single UVB dose (312 nm, 5 kJ/m^2^) using the Stratalinker apparatus (Stratagene). This dose corresponds to the minimal erythema dose (MED) of these mice, inducing erythema 24 h later.

### Ethics statement

The present animal study follows the 3R legislation (Replace-Reduce-Refine). It has been declared and approved by the French Government Board (N°5347). Animal welfare is a constant priority: animals were thus sacrificed under anesthesia.

### Cell culture, small hairpin (sh) RNA transductions, and inhibitor treatments

Mice primary fibroblasts were isolated by collagenase dissociation of skin dermis from *Usf1* ^-/-^ and *Usf1* ^+/+^ mice [74]. Cells were cultured in DMEM (Invitrogen) medium containing 10% FBS and 1% penicillin-streptomycin at 37°C under a 5% CO_2_ atmosphere for one week before the irradiation protocol. Cells were then irradiated with 0.6 kJ/m^2^ UVB and harvested at the indicated time points.

B16 mice melanoma cells were transduced with lentiviral particles containing a vector carrying an shRNA (Sigma) targeting the murine *Usf1* mRNA (sh-*Usf1* SHCLNV-NM_009480 clone TRCN0000302005) or the *Trp53* mRNA (sh-*Trp53*; SHCLNV-NM_011640 clone TRCN000030210844), or carrying scrambled shRNA (sh-*CT* (SHC002V). After infection, cells were maintained under selection in the presence of puromycin (Invitrogen). Cells were then routinely cultured in RPMI (Invitrogen) supplemented with 10% FBS and 1% penicillin-streptomycin at 37°C under a 10% CO_2_ atmosphere. Cell were irradiated with 0.3 kJ/m^2^ UVB and harvested at the indicated time points. For MG132 assays, cells were treated with 10 µM Z-Leu-Leu-al (Sigma) in RPMI (Invitrogen) medium. For cycloheximide (CHX) treatment, after 3 h of MG132 treatment the culture medium was removed and replaced by medium containing 20 µM CHX (Sigma). For Nutlin-3 treatments, cells were stimulated with 10 µM of Nutlin-3 (Santa cruz).

### Cell cycle synchronization, cell viability and BrdU incorporation analysis

B16 melanoma cells were synchronized in G1/S phase following a double thymidine/aphidicolin block (16 h with 2 mM thymidine, released for 9 hours and then 16 h with 5 µg/ml aphidicolin).Cell viability following exposure to UV was measured using MTT test as previously described [21].

BrdU analysis was carried using an *in situ* BrdU detection kit (BD Biosciences): as recommended by the manufacturer. Positively stained cells (BrdU positives) and total cells (hematoxylin stained) in 10 randomly selected microscopic fields (x100) were counted for each condition.

### Gene expression analysis

RNA extraction and RT-PCRq were as previously described [21]. Relative amounts of transcripts were determined using the delta Ct method. Data were normalized to the values for the *HPRT* transcript. Forward (F) and reverse (R) primers were designed using the Universal Probe Library Assay Design Center (Roche) and their efficiency has been confirmed.

### Western blotting and immunoprecipitation

Mouse skin proteins were extracted by pottering 8 mm diameter skin biopsies in liquid nitrogen; the resulting powders were lysed in a lysis buffer containing 10 mM EDTA, 50 mM pH 8 Tris-HCl, 0.5% Empigen BB, 1% SDS, 25 mM NaF, 1 mM orthovanadate, 25 mM β-glycerophosphate, and 1x protease inhibitor cocktail (Roche Diagnostics). Cell culture protein lysates were obtained by scraping off cells in NP40 lysis buffer: 20 mM Tris pH 7.5, 100 mM NaCl, 20 mM β-glycerophosphate, 5 mM MgCl_2_, 0.2% NP-40, 10% glycerol, 1 mM NaF, 0.5 mM DTT, 1x protease inhibitor cocktail. Equal amounts of protein (30 µg), quantified using the BCA protein assay (Sigma) were denaturated in Laemmli buffer for 5 min at 95°C and resolved by 15% SDS-PAGE. Membranes were probed with appropriate antibodies and signals detected using the LAS-3000 Imaging System (Fujifilm) were quantified with ImageJ (http://rsbweb.nih.gov/ij/). The following primary antibody were used: anti-USF1 (C:20), anti-HSC70 (B-6), anti-MDM2 (SMP14), anti-GFP (Santa Cruz), anti-CPD (TDM2) (MBL), anti-p53 (1C12), anti-phospho H2AX Ser139 (γH2AX) (Cell Signaling), anti-total histone H2AX, anti-p21 (Abcam), and anti-MDM2 (3G9) (Millipore) and anti-Ubiquitin (Dako).

Co-immuno-precipitation experiments were performed using 1 mg of protein with 2 µg of Rabbit IgG (Jackson ImmunoResearch, West Grove, PA) as negative control or with 2 µg of USF1 antibodies (C:20) or MDM2 antibodies (SMP14 and 3G9) and incubated overnight at 4°C. Flag-tag proteins were immunoprecipitated using the flag immunoprecipitation kit (Sigma). Immunocomplexes were isolated using Protein A-G sepharose beads.

### Luciferase activity and transitory transfections

To analyze the transcriptional activity of p53, B16 melanoma cells in 10 cm-diameter dishes were transiently transfected with 5 µg of pG13-Luc (carrying a p53-responsive element; [75]) alone or in combination with 6 µg of pCMV GFP (encoding the GFP) or pCMV-USF1 (WT, T153E, T153A, AUSF (negative dominant; [15]), or pCAG3.1 (encoding p53; [76]) and incubated for 24 h. Cells were then passaged in 12-well plates and irradiated 24 h post passage (a total of 48 h post transfection). Luciferase analysis was conducted using the DUAL-Luciferase reporter assay kit according to the manufacturer's recommendations (Promega).

To study p53 degradation in the presence of MDM2 B16 melanoma cells in 6-well plates were transfected with 1 µg of a plasmid encoding flag-tagged p53 protein (Flag-p53/pRK5; Addgen, Plasmid 39237) alone or in combination with 2 µg of a plasmid encoding the MDM2 protein (pCMV-myc3-HDM2; Addgen, Plasmid 20935). For p53 stabilization rescue analysis in sh-*Usf1* KD cells, cells were co-transfected with 2 µg of plasmid encoding GFP protein or USF1 wild type protein [15], together with 1 µg of Flag-p53/pRK5 plus 2 µg of pCMV-myc3-HDM2. The amount of plasmid DNA used for transfection was adjusted with empty pCMV plasmid to be equal in every case.

### CPD quantification by ELISA

CPD in skin tissues was assayed by ELISA, according to Cosmo bio recommendations (Cosmo Bio Co., LTD., Japan). DNA was purified with Nucleospin tissue extraction kits (Macherey-Nagel, Düren, Germany). Briefly, 200 ng aliquots of denatured DNA were distributed into 96-well plates precoated with protamine sulfate (Polyvinylchloride flat-bottom). DNA lesions were detected with specific mouse anti-CPD antibodies [21], and bound antibody was revealed with a biotin/peroxidase-streptovidin system. The absorbance at 492 nm was used for quantitative measurements.

### Immuno-histochemistry and immunofluorescence staining

Skin biopsies were fixed in formalin (4%) and embedded in paraffin. Paraffin-embedded tissu was cut into 4 µm-thick slices, mounted on slides and dried at 58°C for 60 minutes. A Discovery Automated IHC stainer and the Ventana DABMap detection kit (Ventana Medical Systems) were used for immunohistochemical staining. For DAB, detection slides were incubated with rabbit monoclonal anti-CPD antibody (TDM, MBL) and bound antibody was revealed with biotinylated goat anti-rabbit secondary antibody (Vector laboratory, USA). Slides were then counterstained for 4 minutes with hematoxylin and rinsed. For fluorescence analysis, slides were incubated with rabbit monoclonal Ki-67 (SP6, bioscience leasanton CA) and bound antibody was detected with secondary anti-rabbit FITC-conjugated antibody. Slides were coverslipped in aqueous mounting medium containing DAPI. The numbers of Ki-67-positive cells were evaluated by counting the percentage of interfollicular-positive cells (Ki-67 among Dapi-stained cells) in 10 different microscope fields (x40) per skin section.

For cyto-immunofluorecence staining, cells grown on coverslip chambers were fixed with formalin (4%) for 20 min, washed with PBS, quenched with 50 mM NH4Cl for 20 min and washed once with PBS. The cells were permabilized with 0.1% Triton and saturated with 1% PBS/BSA; 15 min later, the primary antibody was added. Bound antibody was reveled with anti-rabbit Alexa 488 or anti-mouse FITC 588 secondary antibodies (Jackson). Experiments have been done in triplicate and 15 to 20 microscopic fields analyzed per condition. A minimum of one hundered cells were analyzed per condition.

### Detection of protein interactions with Duolink using PLA technology (Sigma)

B16 melanoma were transiently or not transfected with pCAG3.1 (encoding p53; [76]), pCMV-MDM2 (encoding MDM2 protein), and pCMV-GFP (encoding the GFP protein) or various pCMV-USF1 expressing vector (WT and AUSF forms; [15]), and incubated for 24 h. Cells were then passaged in 96-well plates and fixed using PFA 24 h post passage (a total of 48 h post transfection). Protein-protein (USF1 and p53) or (p53 and MDM2) interactions in B16 melanoma cells were then analyzed following recommanded protocol by manufacturer (Sigma Aldrich) and visualized in collaboration with the ImPACcell plateform (SFR Biosit, University of Rennes, France) using Thermo Scientific Cellomics HCS Solution. For quantification, a minimum of 15 microscopic fields were analyzed and the signal were counted in a minimum of 60 cells. The following primary antibody were used: rabbit anti-USF1 (C:20), mouse anti-MDM2 and mouse anti-p53 (1C12) or rabbit anti-p53 (Fl-393).

## Supporting Information

Figure S1 Loss of USF1 alters skin CPD lesions removal and cell proliferation after UVB irradiation of skin punch biopsies. (A) Level of p53, in *Usf1* ^+/+^ and *Usf1* ^-/-^ mice skin-exposed areas versus non-irradiated areas (controls),12 hours post irradiation. Western blot showing USF1, p53, γH2AX and HSC70 (loading control) immunoreactivity 12 h after skin irradiated or not irradiated with UVB. Graph reports the mean ratio between the p53 signal (normalized to that for HSC70). Error bars: SD, n = 5 for each condition. (B) *Usf1^+/+^* (*Usf1* WT) and *Usf1^-/-^* (*Usf1* KO) cultured skins explants were or were not irradiated with UVB (5 kJ/m^2^) and analyzed for the induction of transcripts *ex vivo.* RT-qPCR analysis of *CDKN1a* (p21), *SFN* (14-3-3σ) and *PCNA* transcripts in UVB-irradiated skin and non-exposed controls; values reported were normalized to those for the *Hprt* transcript. Transcripts were assayed *in vivo* 5 hours after irradiation. Error bars: SD, n = 3 *ex vivo*. (C) Detection of CPD DNA-damage by immunostaining microscopy (x100) in skin punch biopsies from WT (*Usf1^+/+^*) (left panel) or *Usf1* KO mice (*Usf1^-/-^*) (right panel) before and after irradiation (ranging from 3 to 24 hours) of skin with 5 kJ/m^2^ of UVB. (D) *Ex vivo* analysis by ELISA quantification of CPD (using specific anti-CPD antibody (CosmoBio LTD.)) kinetic of removal (ranging from 3 to 24 hours) in WT and KO mice skin biopsies treated with 5 kj/m^2^ UVB. Graph represents the mean of CPD content in DNA extracted from exposed skin at different times, the experiments was performed two times in duplicate. (E) *Ex vivo* analysis of Ki-67 skin-interfolliclar staining in skin biopsies of WT and KO mice dorsal skin treated with 5 kj/m^2^ UVB and harvested after different times (ranging from 3 to 24 hours). Graph representing the quantification of interfollicular Ki-67 stained cells in UVB exposed skin cultures, data are expressed as percentage of stained cells compared to non-exposed skin controls.
(JPG)

Figure S2 USF1 KO fibroblasts override S phase arrest following genotoxic stress. Primary fibroblasts isolated from *Usf1^+/+^* and *Usf1^-/-^* mice were analyzed for S phase progression, and regulation of p53 and p21 following UVB irradiation (0.6 k/jm^2^). (A) Graph reporting the mean percentage of primary fibroblasts incorporating BrdU after irradiation (0.6 k/jm^2^); values for non-irradiated controls are given for reference. Error bars: SD, n = 3. (B) MTT activity evaluation of primary fibroblast viability after UVB irradiation compared to non-irradiated controls treated as in A. Error bars: SD, n = 3. (C) Western blot analysis of p53 and p21 in primary fibroblasts 6 hours after UVB irradiation. The graph represents the densitometric evaluation of p21 and p53 bands (normalized to those for HSC70). Error bars: SD, n = 3.
(JPG)

Figure S3 USF1 is required to promote p53 activity. B16 melanoma cells knocked down for *Usf1* were tested for their ability to modulate p53 level and specific activity in response to UVB irradiation (6 h after 0.3 kJ/m^2^). (A) Western blot analysis of p53, p21 and HSC70 (loading control) proteins in sh-*CT,* sh-*Usf1* and sh-*Trp53* cells following UVB irradiation. (B) p53 transcriptional activity in sh-*CT,* sh-*Usf1* and sh-*Trp53* cells transfected with a reporter plasmid encoding a p53 responsive element (p53-RE) driving the luciferase gene and irradiated or not irradiated with UVB. The graph reports luciferase activity following UVB irradiation with the values for non-irradiated sh-*CT* cells used for reference. Error bars: SD, n = 3. (C) Same experiment as in B but with sh-*Usf1* KD cells co-transfected with a reporter plasmid encoding a p53 responsive element together with GFP or different USF1 cDNA constructs. Schematic representation of the USF1 protein (with its DNA-Binding grey square, HLH light grey square and LZ dark grey square domains) and various point mutations modulating USF1 transcriptional activity: positively (T153E) or negatively (T153A) and deletion form lacking DNA-binding domain and transcriptional activity (AUSF). Error bars: SD, n = 3. (D) Western blotting analysis of protein extracted of skin from WT mice (*Usf1^+/+^*) and *Usf1* KO mice (*Usf1^-/-^*) irradiated or not irradiated with UVB (5 kJ/m^2^) analyzed 5 h later. (E) Western blotting analysis of protein extracted from B16 melanoma cells knocked down for *Usf1* (sh-*Usf1*) or control cells (sh-*CT*) irradiated or not irradiated with increasing doses of UVB (0 to 1.5 kJ/m^2^) and analyzed 5 h later. Western blots show USF1, P-CHK1, γH2AX, p53 and HSC70 (loading control) immunoreactivity after or not UVB irradiation.
(JPG)

Figure S4 p53 and MDM2 stability in response to UV in sh-*Usf1* cells. (A) p53 degradation in sh-*CT* and sh-*Usf1* cells pretreated for 3 h with MG132 (10 µM) and then treated with UVB previously to cycloheximide (CHX 20 µM). Cells were analyzed at the time points indicated after UVB. The graphs show the results of densitometric analysis of p53 immunoreactive bands (normalized to the loading controls H2AX or HSC70). (B) Western blot showing MDM2 and αTub immunoreactivity in B16 melanoma cells knocked down for *Usf1* (sh-*Usf1*) or control cells (sh-*CT*) cells at the indicated time following treatment with MG132 (10 µM). (C-D) Time course of MDM2 accumulation in sh-*CT* and sh-*Usf1* cells treated with vehicle (DMSO) in C or MG132 (10 µM) plus UVB (0.3 kJ/m^2^) irradiation in D. The graphs show the results of densitometric analysis of MDM2 immunoreactive bands (normalized to the loading controls αTub).
(JPG)
