# Supplementary figures and images for: p53 Requires the Stress Sensor USF1 to Direct Appropriate Cell Fate Decision
Source: PLoS Genet. 2014 May 15;10(5):e1004309. doi: 10.1371/journal.pgen.1004309 (PMC4022457; doi:10.1371/journal.pgen.1004309)

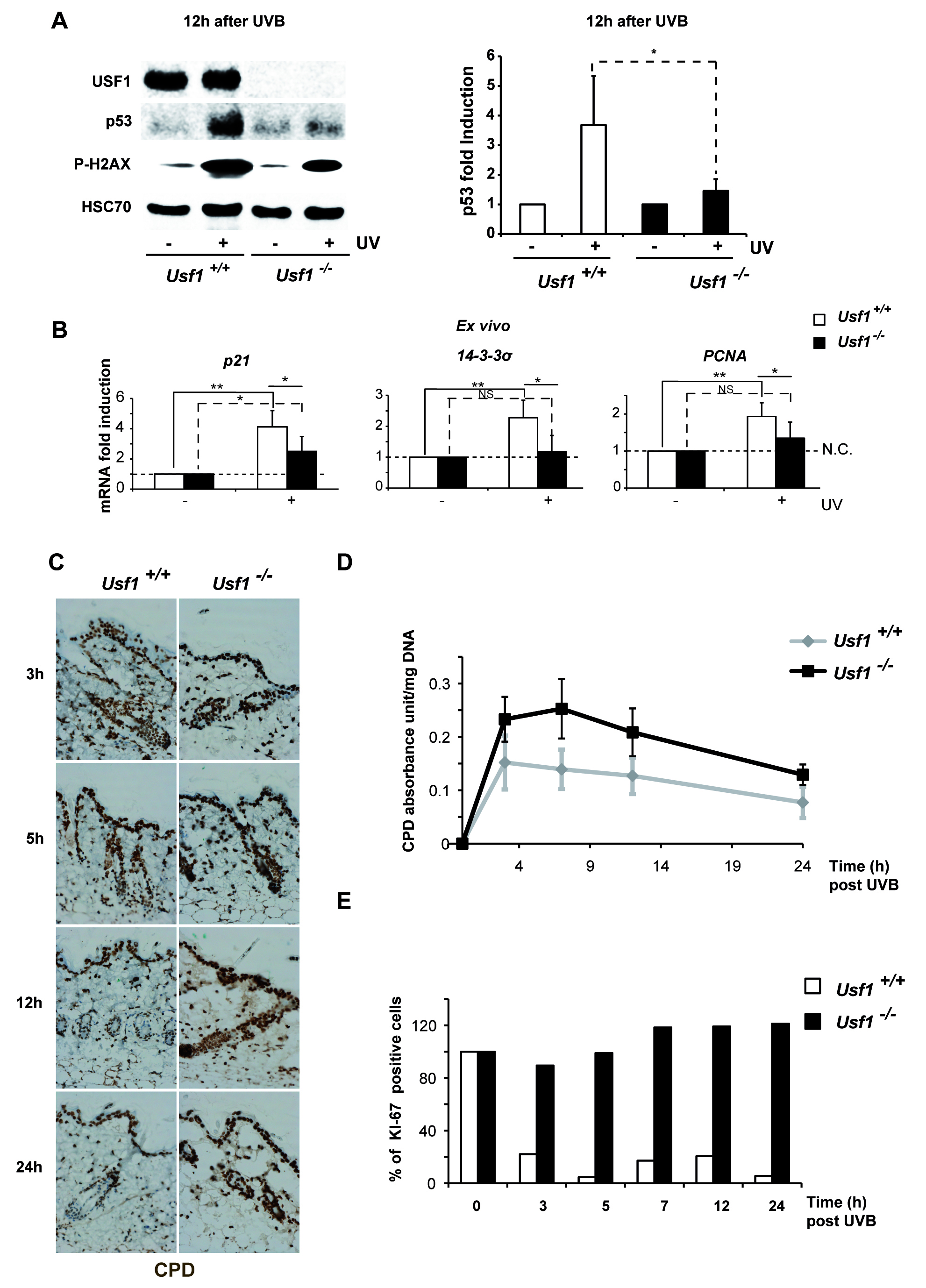

Supplement: Figure S1 — Loss of USF1 alters skin CPD lesions removal and cell proliferation after UVB irradiation of skin punch biopsies. (A) Level of p53, in Usf1 +/+ and Usf1 -/- mice skin-exposed areas versus non-irradiated areas (controls),12 hours post irradiation. Western blot showing USF1, p53, γH2AX and HSC70 (loading control) immunoreactivity 12 h after skin irradiated or not irradiated with UVB. Graph reports the mean ratio between the p53 signal (normalized to that for HSC70). Error bars: SD, n = 5 for each condition. (B) Usf1+/+ (Usf1 WT) and Usf1-/- (Usf1 KO) cultured skins explants were or were not irradiated with UVB (5 kJ/m2) and analyzed for the induction of transcripts ex vivo. RT-qPCR analysis of CDKN1a (p21), SFN (14-3-3σ) and PCNA transcripts in UVB-irradiated skin and non-exposed controls; values reported were normalized to those for the Hprt transcript. Transcripts were assayed in vivo 5 hours after irradiation. Error bars: SD, n = 3 ex vivo. (C) Detection of CPD DNA-damage by immunostaining microscopy (x100) in skin punch biopsies from WT (Usf1+/+) (left panel) or Usf1 KO mice (Usf1-/-) (right panel) before and after irradiation (ranging from 3 to 24 hours) of skin with 5 kJ/m2 of UVB. (D) Ex vivo analysis by ELISA quantification of CPD (using specific anti-CPD antibody (CosmoBio LTD.)) kinetic of removal (ranging from 3 to 24 hours) in WT and KO mice skin biopsies treated with 5 kj/m2 UVB. Graph represents the mean of CPD content in DNA extracted from exposed skin at different times, the experiments was performed two times in duplicate. (E) Ex vivo analysis of Ki-67 skin-interfolliclar staining in skin biopsies of WT and KO mice dorsal skin treated with 5 kj/m2 UVB and harvested after different times (ranging from 3 to 24 hours). Graph representing the quantification of interfollicular Ki-67 stained cells in UVB exposed skin cultures, data are expressed as percentage of stained cells compared to non-exposed skin controls. (JPG) [file pgen.1004309.s001.jpg]

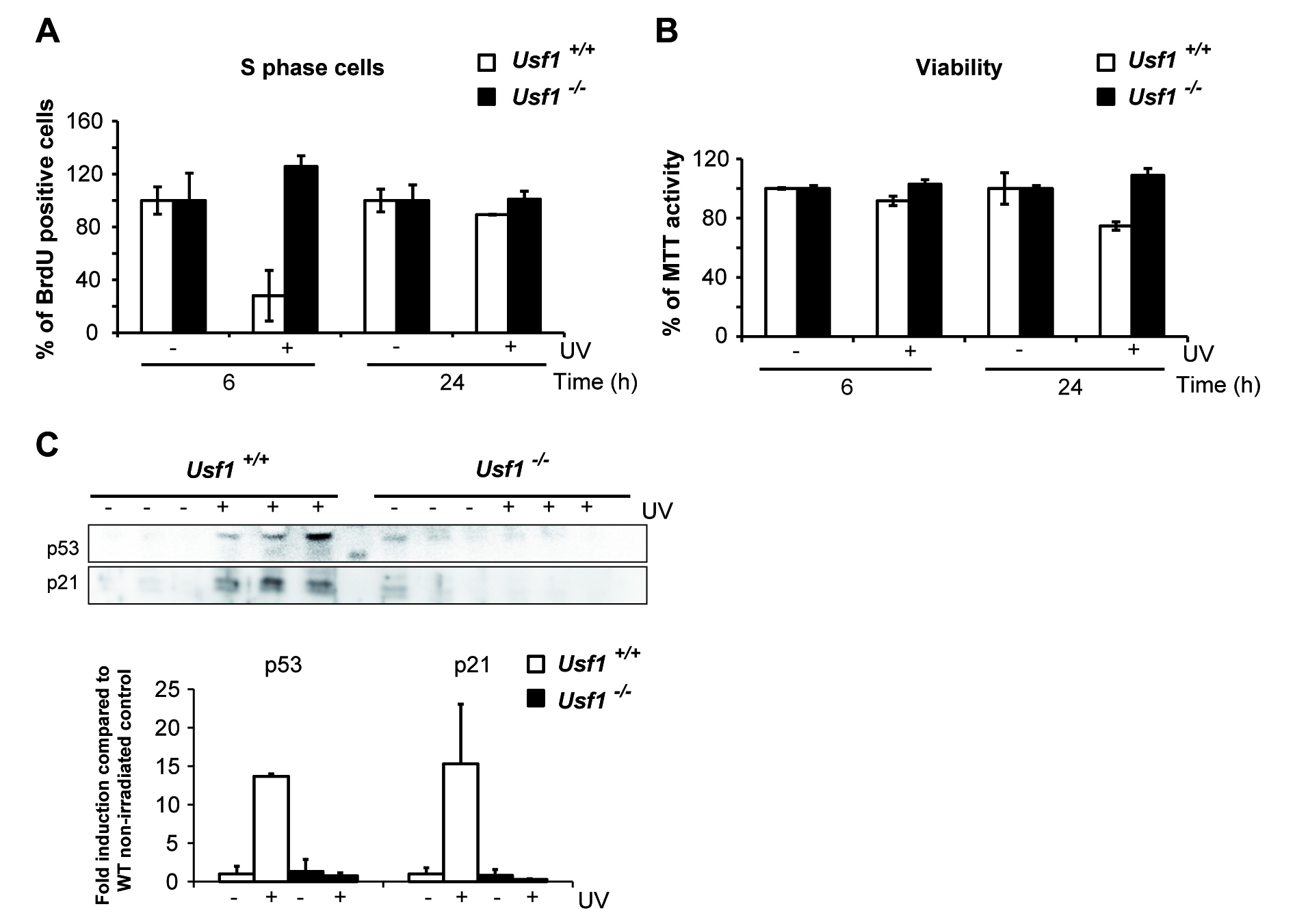

Supplement: Figure S2 — USF1 KO fibroblasts override S phase arrest following genotoxic stress. Primary fibroblasts isolated from Usf1+/+ and Usf1-/- mice were analyzed for S phase progression, and regulation of p53 and p21 following UVB irradiation (0.6 k/jm2). (A) Graph reporting the mean percentage of primary fibroblasts incorporating BrdU after irradiation (0.6 k/jm2); values for non-irradiated controls are given for reference. Error bars: SD, n = 3. (B) MTT activity evaluation of primary fibroblast viability after UVB irradiation compared to non-irradiated controls treated as in A. Error bars: SD, n = 3. (C) Western blot analysis of p53 and p21 in primary fibroblasts 6 hours after UVB irradiation. The graph represents the densitometric evaluation of p21 and p53 bands (normalized to those for HSC70). Error bars: SD, n = 3. (JPG) [file pgen.1004309.s002.jpg]

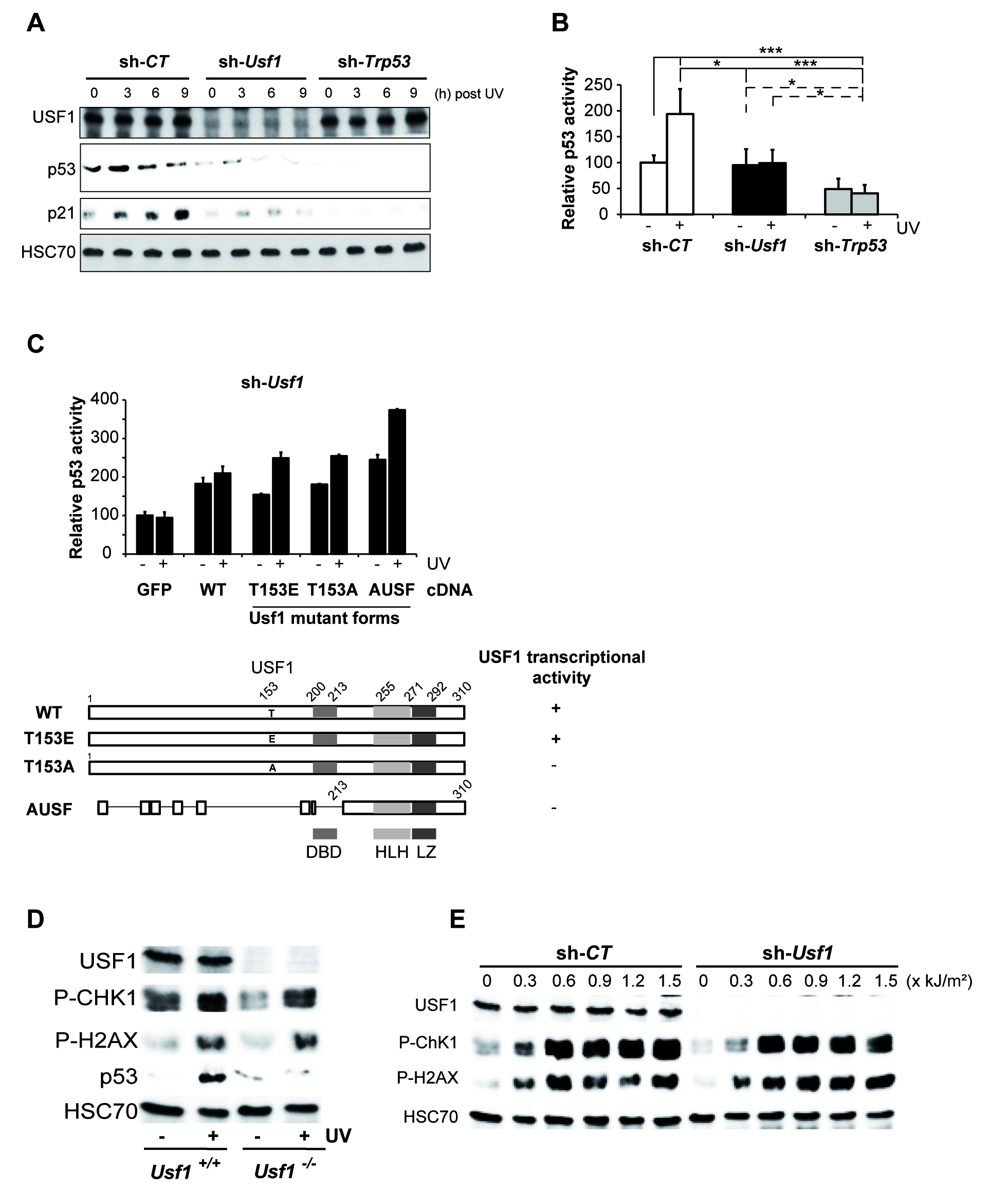

Supplement: Figure S3 — USF1 is required to promote p53 activity. B16 melanoma cells knocked down for Usf1 were tested for their ability to modulate p53 level and specific activity in response to UVB irradiation (6 h after 0.3 kJ/m2). (A) Western blot analysis of p53, p21 and HSC70 (loading control) proteins in sh-CT, sh-Usf1 and sh-Trp53 cells following UVB irradiation. (B) p53 transcriptional activity in sh-CT, sh-Usf1 and sh-Trp53 cells transfected with a reporter plasmid encoding a p53 responsive element (p53-RE) driving the luciferase gene and irradiated or not irradiated with UVB. The graph reports luciferase activity following UVB irradiation with the values for non-irradiated sh-CT cells used for reference. Error bars: SD, n = 3. (C) Same experiment as in B but with sh-Usf1 KD cells co-transfected with a reporter plasmid encoding a p53 responsive element together with GFP or different USF1 cDNA constructs. Schematic representation of the USF1 protein (with its DNA-Binding grey square, HLH light grey square and LZ dark grey square domains) and various point mutations modulating USF1 transcriptional activity: positively (T153E) or negatively (T153A) and deletion form lacking DNA-binding domain and transcriptional activity (AUSF). Error bars: SD, n = 3. (D) Western blotting analysis of protein extracted of skin from WT mice (Usf1+/+) and Usf1 KO mice (Usf1-/-) irradiated or not irradiated with UVB (5 kJ/m2) analyzed 5 h later. (E) Western blotting analysis of protein extracted from B16 melanoma cells knocked down for Usf1 (sh-Usf1) or control cells (sh-CT) irradiated or not irradiated with increasing doses of UVB (0 to 1.5 kJ/m2) and analyzed 5 h later. Western blots show USF1, P-CHK1, γH2AX, p53 and HSC70 (loading control) immunoreactivity after or not UVB irradiation. (JPG) [file pgen.1004309.s003.jpg]

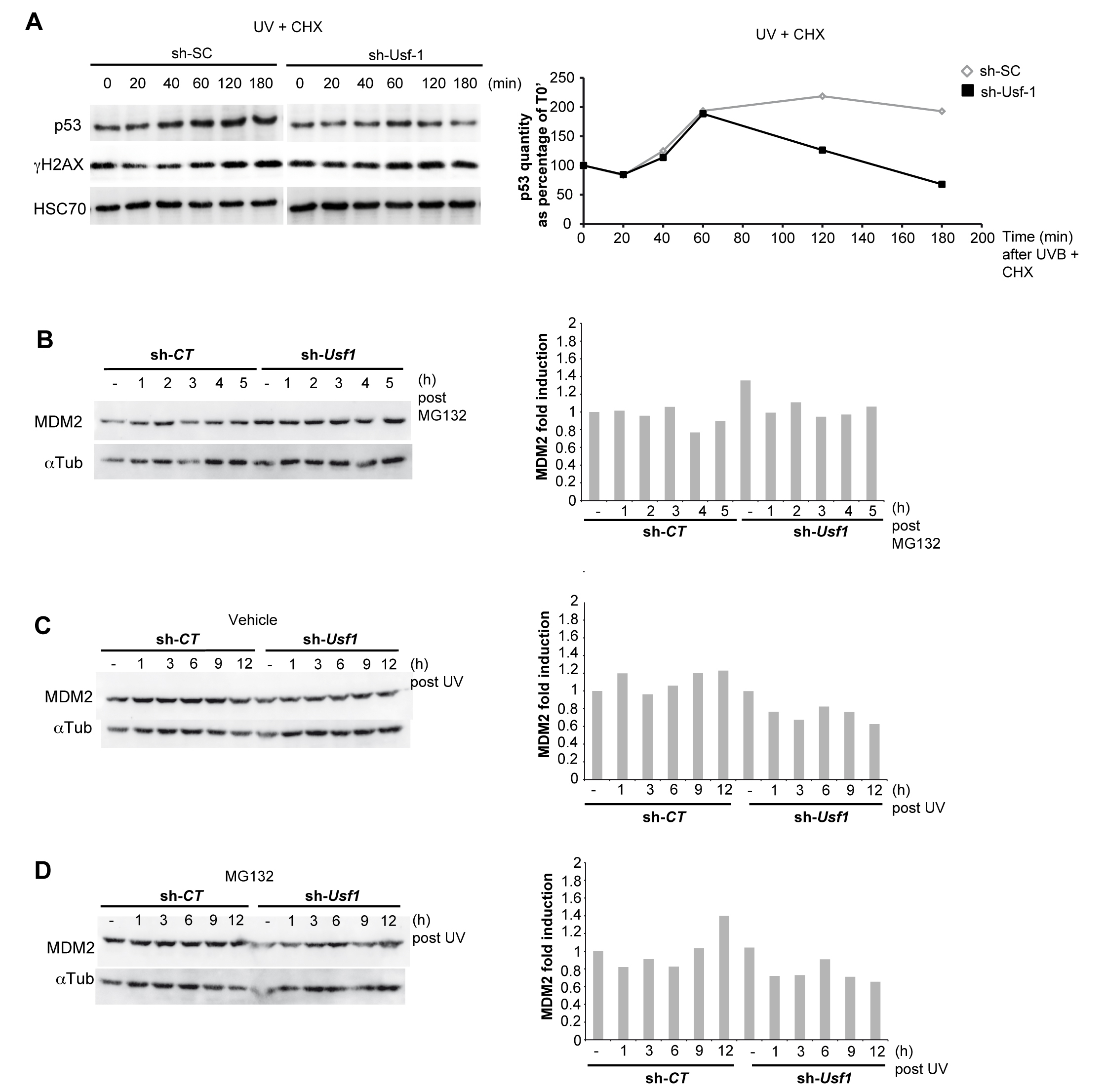

Supplement: Figure S4 — p53 and MDM2 stability in response to UV in sh-Usf1 cells. (A) p53 degradation in sh-CT and sh-Usf1 cells pretreated for 3 h with MG132 (10 µM) and then treated with UVB previously to cycloheximide (CHX 20 µM). Cells were analyzed at the time points indicated after UVB. The graphs show the results of densitometric analysis of p53 immunoreactive bands (normalized to the loading controls H2AX or HSC70). (B) Western blot showing MDM2 and αTub immunoreactivity in B16 melanoma cells knocked down for Usf1 (sh-Usf1) or control cells (sh-CT) cells at the indicated time following treatment with MG132 (10 µM). (C-D) Time course of MDM2 accumulation in sh-CT and sh-Usf1 cells treated with vehicle (DMSO) in C or MG132 (10 µM) plus UVB (0.3 kJ/m2) irradiation in D. The graphs show the results of densitometric analysis of MDM2 immunoreactive bands (normalized to the loading controls αTub). (JPG) [file pgen.1004309.s004.jpg]
